# Conditional Knockout of Breast Carcinoma Amplified Sequence 2 (BCAS2) in Mouse Forebrain Causes Dendritic Malformation via β-catenin

**DOI:** 10.1038/srep34927

**Published:** 2016-10-07

**Authors:** Chu-Wei Huang, Yi-Wen Chen, Yi-Rou Lin, Po-Han Chen, Meng-Hsuan Chou, Li-Jen Lee, Pei-Yu Wang, June-Tai Wu, Yeou-Ping Tsao, Show-Li Chen

**Affiliations:** 1 Graduate Institute of Microbiology, College of Medicine, National Taiwan University, Taipei 100, Taiwan; 2 Graduate Institute of Anatomy and Cell Biology, College of Medicine, National Taiwan University, Taipei 100, Taiwan; 3 Graduate Institute of Brain and Mind Sciences, College of Medicine, National Taiwan University, Taipei 100, Taiwan; 4 Institute of Molecular Medicine, College of Medicine, National Taiwan University, Taipei 100, Taiwan; 5 Department of Medical Research, National Taiwan University Hospital, Taipei 100, Taiwan; 6 Department of Ophthalmology, Mackay Memorial Hospital, Taipei 104, Taiwan

## Abstract

Breast carcinoma amplified sequence 2 (BCAS2) is a core component of the hPrP19 complex that controls RNA splicing. Here, we performed an exon array assay and showed that β-catenin is a target of BCAS2 splicing regulation. The regulation of dendrite growth and morphology by β-catenin is well documented. Therefore, we generated conditional knockout (cKO) mice to eliminate the BCAS2 expression in the forebrain to investigate the role of BCAS2 in dendrite growth. BCAS2 cKO mice showed a microcephaly-like phenotype with a reduced volume in the dentate gyrus (DG) and low levels of learning and memory, as evaluated using Morris water maze analysis and passive avoidance, respectively. Golgi staining revealed shorter dendrites, less dendritic complexity and decreased spine density in the DG of BCAS2 cKO mice. Moreover, the cKO mice displayed a short dendrite length in newborn neurons labeled by DCX, a marker of immature neurons, and BrdU incorporation. To further examine the mechanism underlying BCAS2-mediated dendritic malformation, we overexpressed β-catenin in BCAS2-depleted primary neurons and found that the dendritic growth was restored. In summary, BCAS2 is an upstream regulator of β-catenin gene expression and plays a role in dendrite growth at least partly through β-catenin.

The breast carcinoma amplified sequence 2 (BCAS2) gene is mapped to the human chromosome 1p13.2 region encoding a 26-kD small nuclear protein. BCAS2 binds to the estrogen receptor (ER) to mediate ER gene expression^1^,^2^. BCAS2 is also involved in prostate cancer through interactions with the androgen receptor^3^. Recent studies demonstrated at least 2 BCAS2 mechanisms associated with carcinogenesis: (1) negative regulation of p53: depletion of BCAS2 leads to apoptosis in p53 wild-type cell lines but induces G2/M growth arrest in p53 null and p53 mutant cells^4^; and (2) binding to a single-stranded DNA-binding protein involved in the DNA repair function of the hPrp19 complex^5^, which participates in the DNA damage response^6^.

BCAS2 is a core member of the Prp19 spliceosome complex to regulate RNA splicing^7^,^8^. Knockdown of the BCAS2/spf27 subunit destabilizes the hPrp19 complex, which results in mitotic defects^9^. BCAS2 was recently found to be an essential gene during the embryonic stage^10^. We previously reported that wing-specific knockdown of BCAS2 causes *Drosophila* wing developmental defects^11^; due to BCAS2 participates in Delta pre-mRNA splicing and regulates the Delta-Notch signaling^12^. Delta-Notch signaling is known to be important in neurogenesis. For example, mice deficient in *Dlk1* show defects in postnatal neurogenesis within the subventricular zone (SVZ), which results in a lack of mature neurons in the olfactory bulb^13^. To gain insight into BCAS2-targeted genes, we performed exon array analysis using BCAS2-depleted cells. From the functional catalogue of alternative splicing events, β-catenin was a candidate for BCAS2-targeted genes (Supplementary Table S1). Wnt/β-catenin signaling plays an important role in neuronal development and neurogenesis^14^,^15^. Disruption of β-catenin results in dendritic and brain malformation, leading to degenerative diseases^16^,^17^,^18^.

Neurons in the mammalian brain contain complicated dendritic trees. Dendrites are important for synaptic connections that are modified by the intensity and frequency of synaptic activity. Therefore, dendritic development is critical for neural circuit formation and synaptic input processing for cognitive processes such as learning and memory^19^. Global KO of BCAS2 is lethal^10^, and this finding is consistent with current reports showing that depleting one member of the hPrp19 complex (e.g., PLGR1 or PSO4) leads to early embryonic lethality in a mouse model^20^,^21^. To study the role of BCAS2 in dendritic development, we crossed our floxed-BCAS2 mice with calcium/calmodulin-dependent protein kinase IIα (CaMKIIα)-iCre transgenic mice to conditionally knockout BCAS2 in the postnatal forebrain. Conditional knockout (cKO) of BCAS2 in the forebrain resulted in a microcephaly-like brain size. BCAS2 cKO mice exhibited impaired learning and memory and dendritic malformation in the hippocampus. Additionally, depletion of BCAS2 reduced the splicing efficiency of β-catenin pre-mRNA in cell lines and cKO mice. Moreover, exogenous expression of β-catenin could restore the reduced dendritic length in the primary neuronal cells. In summary, BCAS2 is an upstream regulator of β-catenin gene expression, and it plays a role in dendrite growth at least partly through β-catenin.

## Results

### Expression of β-catenin is regulated by BCAS2 via pre-mRNA splicing

**Figure 1:**
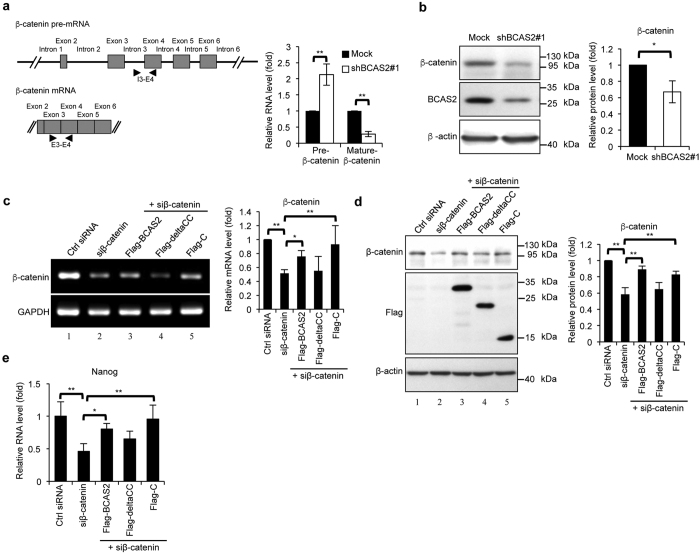
BCAS2 regulates β-catenin gene expression. (**a**) BCAS2 knockdown in MCF7 cells increases β-catenin pre-mRNA and reduces β-catenin mRNA levels. Left: schematic representation of the design of primers to detect the intron-containing precursor mRNA (upper) and mRNA of β-catenin (lower). The locations of primers, exons and introns are denoted with arrowheads, boxes and lines, respectively. Right: quantitative RT-PCR analysis (n = 3). (**b**) Decreased β-catenin protein in BCAS2-knockdown MCF7 cells. Left, a representative western blot. Right, quantification of the left panel (n = 3). (**c,d**) Full-length BCAS2 and C-terminal of BCAS2 increased the β-catenin RNA (**c**) and protein (**d**) expression in siβ-catenin-treated N2A cells, but deltaCC did not affect the β-catenin expression. The full-length BCAS2, C-fragment, and deltaCC mutant plasmids were transfected into the β-catenin-silenced N2A cells separately. Forty-eight hours after transfection, the RNA and protein were extracted for RT-PCR and western analysis, respectively. Left: representative result. Right: quantitation of left panel (n = 3). (**e**) qRT-PCR showing *nanog* expression (n = 3). Panels a, b: The *P* value was analyzed by a two-tailed Student’s *t*-test. Panels c, d, and e: one-way ANOVA. Results are shown as the mean ± S.D. Uncropped blots are presented in Supplementary Fig. S5.

BCAS2 regulates pre-mRNA splicing^7^,^11^. To search for candidate BCAS2-targeted genes, we extracted RNA from BCAS2-knockdown MCF7 cells to perform exon array analysis (Supplementary Table S1) and found that β-catenin was a potential BCAS2-targeted gene. To confirm whether the efficiency of β-catenin splicing could be affected by BCAS2 depletion, we evaluated the expression of β-catenin mRNA and pre-mRNA. The results showed a significant reduction in the mRNA level of β-catenin and an accumulation of β-catenin pre-mRNA in BCAS2-depleted MCF7 cells (Fig. 1a, right), indicating that the splicing efficiency was impaired in the absence of BCAS2. Moreover, we confirmed the decreased protein expression of β-catenin in BCAS2-depleted MCF7 cells (Fig. 1b). Previously, we found that the BCAS2 C-terminus containing the coiled-coil motifs is essential for RNA splicing. The full-length or C fragment of BCAS2 is functional for RNA splicing, but the deltaCC mutant (deleted coiled-coil motifs) cannot perform its splicing role^11^. To further confirm whether BCAS2 can regulate β-catenin splicing, we transfected full-length BCAS2, the deltaCC mutant or the C fragment into β-catenin-silenced N2A cells and then measured the RNA and protein expression by RT-PCR and western blot, respectively. Because of the high β-catenin expression in N2A cells (Fig. 1c, lane 1), the forced BCAS2 expression could not increase the β-catenin RNA and protein levels (data not shown). Therefore, we needed to generate a low β-catenin expression environment to analyze the effects of BCAS2 mutants on β-catenin splicing. We treated N2A cells with siβ-catenin to knock down the β-catenin RNA (Fig. 1c, lane 2) and protein (Fig. 1d, lane 2). Overexpression of BCAS2 and the C fragment increased the β-catenin RNA and protein expression (lanes 3 and 5 of Fig. 1c,d) compared with that in the low-β-catenin level N2A cells treated with siβ-catenin (lane 2 of Fig. 1c,d); in contrast, the deltaCC mutant had no effect (lane 4 of Fig. 1c,d). Furthermore, *nanog*, a β-catenin-targeted gene^22^,^23^, was still decreased in deltaCC-transfected β-catenin silenced cells, but overexpression of the full-length or C fragment of BCAS2 rescued the mRNA expression of *nanog* (Fig. 1e). Taken together, these results show that BCAS2 can regulate β-catenin RNA splicing and expression.

### BCAS2 distribution in brain

BCAS2 is involved in β-catenin splicing; thus, depletion of BCAS2 results in a reduction in β-catenin gene expression (Fig. 1). Previous studies demonstrated that Wnt/β-catenin signaling plays an important role in neurogenesis^14^,^16^,^24^. In this study, we investigated the role of BCAS2 in dendrite development in mice. Firstly, to examine the BCAS2 distribution in various tissues of wild-type mice (WT), western blot analysis was performed, and the results showed that BCAS2 was expressed in various tissues, such as the eye, kidney, lung, and brain (Fig. S1a). IHC analysis revealed BCAS2 expression in the entire forebrain, including the cortex, hippocampus, thalamus, hypothalamus and amygdala, of WT mice (Fig. S1b). We then used doublecortin (DCX) and NeuN as immature neuron and mature neuron markers, respectively^25^, to examine the expression pattern of BCAS2 in different neuronal cell types. DCX was expressed in the cytoplasm of immature neurons in the inner cell layer of the DG (Fig. S1c) and was co-expressed with BCAS2. NeuN was expressed in the nuclei of mature neurons in the GCL of the DG and was also co-expressed with BCAS2 (Fig. S1d). Thus, BCAS2 can be expressed in the forebrain in both immature and mature neurons.

### Generation of forebrain-specific BCAS2 KO mice

**Figure 2:**
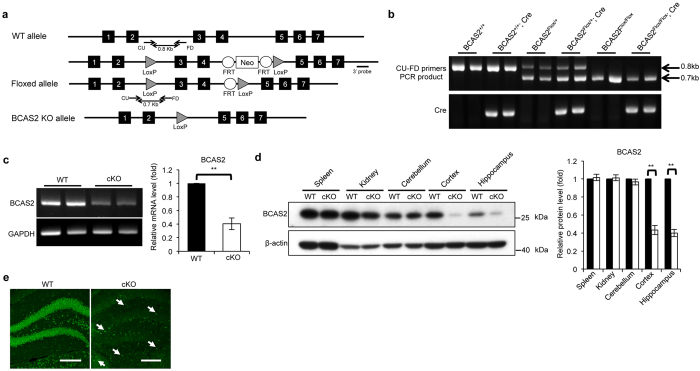
Generation of BCAS2 conditional knockout (cKO) mice. (**a**) Schematic representation of the BCAS2 gene targeting strategy. The LoxP-floxed allele contains one LoxP inserted between exons 2 and 3 and the other LoxP inserted between exons 4 and 5 with two Flip recombinase targets (FRTs) flanking the Neo cassette. The Neo cassette was removed by Flip recombinase. Exons 3 and 4 were deleted by CaMKIIα-iCre recombinase. CU and FD are primers for genotyping. (**b**) Genotyping by PCR. BCAS2^Flox/+^;CaMKIIα-iCre^+^ offspring were crossed with each other, which resulted in 6 different genotypes. Genomic DNA was extracted from tails of offspring. Upper: The sizes of the bands identified by CU and FD primers were 0.8 kb for BCAS2^+/+^, 0.8 and 0.7 kb for the heterozygous BCAS2-floxed allele (BCAS2^Flox/+^), and 0.7 kb for the homozygous BCAS2-floxed allele (BCAS2^Flox/Flox^). Lower: Cre gene. In this study, BCAS2^Flox/Flox^;CaMKIIα-iCre^+^ mice were considered the forebrain-specific BCAS2 cKO mice and were named cKO; the WT mice were BCAS2^+/+^ and contained the Cre gene. (**c**) RT-PCR analysis of the BCAS2 mRNA expression in WT and BCAS2 cKO mice at 12 weeks of age. Left, representative RNA. Right, quantification of the left panel (n = 3). (**d**) Western blot analysis of the BCAS2 protein levels for each genotype at 12 weeks of age. Left, representative western blot. Right, quantification of the left panel (n = 3). (**e**) Immunofluorescence assay (IFA) showing the BCAS2 expression level in 12-week-old WT and cKO mice. White arrow: BCAS2 expression in astrocytes of cKO mice. Scale bar: 200 μm. Uncropped blots are presented in Supplementary Fig. S5.

BCAS2 was highly expressed in the brain of WT mice (Fig. S1a). Ubiquitous depletion of BCAS2 causes embryonic lethality in mice^10^. To investigate the role of BCAS2 in dendritic development, we used the Cre/loxP system to generate floxed-BCAS2 mice (BCAS2^Flox/Flox^), in which exons 3 and 4 of BCAS2 were flanked by loxP. Crossing floxed-BCAS2 mice with CaMKIIα-iCre transgenic mice generated a truncated BCAS2 gene with a frameshift mutation (Fig. 2a). CaMKIIα-iCre is expressed in the forebrain beginning at postnatal day 3, with a peak level at day 15^26^. We then verified both the targeted gene deletion in the genotypes of the offspring by RT-PCR (Fig. 2b, upper) and the presence of the *Cre* gene at 3 weeks of age (Fig. 2b, lower). The ratio of the offspring genotypes (BCAS2^+/+^:BCAS2^Flox/+^:BCAS2^Flox/Flox^) was nearly 1:2:1 (data not shown). The BCAS2 mRNA expression in cKO mice (BCAS2^Flox/Flox^;CaMKIIα-iCre^+^ mice) was greatly reduced, as shown by RT-PCR analysis (Fig. 2c). Western blot analysis showed a dramatic reduction in the BCAS2 protein in the cortex and hippocampus but not in the spleen, kidney or cerebellum (Fig. 2d). However, in CaMKIIα-iCre transgenic mice, Cre recombinase is expressed predominantly in neurons but not in glial fibrillary acidic protein (GFAP) astrocytes^27^,^28^; thus, there was still detectable BCAS2 protein expression in the forebrains of cKO mice, as indicated by RT-PCR and western blot analysis (Fig. 2c,d). IFA also showed that the expression of BCAS2 was reduced in the DG of cKO mice, but some BCAS2 expression was still detected (Fig. 2e; white arrow). Hence, BCAS2 should be expressed in the astrocytes of cKO mice. To confirm this conclusion, IFA analysis was performed, and it revealed the presence of BCAS2 in the GFAP-expressing astrocytes from both cKO (Supplementary Fig. S2b) as WT (Supplementary Fig. S2a) mice. Taken together, the results show that we successfully generated BCAS2 cKO mice and specifically deleted BCAS2 in neurons but not astrocytes in the forebrain.

### BCAS2 cKO in the mouse forebrain causes a microcephaly-like phenotype

**Figure 3:**
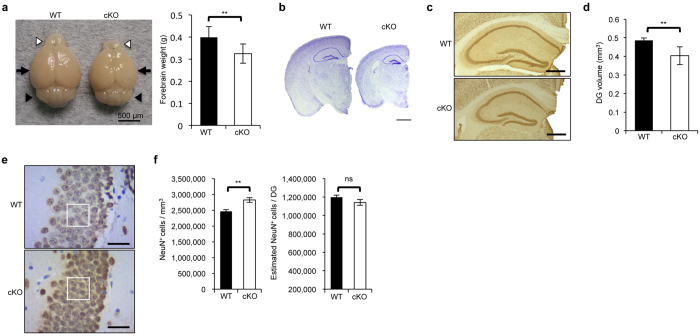
BCAS2 cKO mice show a microcephaly-like phenotype. (**a**) Appearance of brains at 12 weeks of age. Left: gross view of brain. White arrowhead: olfactory bulb; black arrow: forebrain portion; black arrowhead: cerebellum. Right: forebrain weight (WT: n = 11; cKO: n = 16). (**b**) Nissl staining of coronal brain sections. Scale bar: 1 mm. (**c**) IHC with anti-NeuN antibody of representative series of every 12^th^ 40-μm vibratome section. Scale bar: 500 μm. (**d**) DG volume measured by stereological analysis with ImageJ (n = 3). DG volume = (sum of the NeuN^+^ DG areas in every section) × 40 × 12. (**e**) IHC of NeuN^+^ cells. White box: 25 × 25 μm^2^ for counting the cell number, Scale bar: 25 μm. (**f**) Quantification of NeuN^+^ cell density (left) and estimated NeuN^+^ cell number (right) in the DG area. The cell density of granule cell layers in the DG was quantified from 50 randomly chosen counting frames for each genotype (the counting frame is indicated by the white box in panel e) and was then converted to the cell number per cubic millimeter (NeuN^+^ cells/frame area x section thickness, n = 3) multiplied by the DG volume. Quantitative results are shown as the mean ± S.D. and statistically analyzed by a two-tailed Student’s t-test.

We investigated the effect of eliminating BCAS2 in the adult mouse forebrain. Compared with the WT, BCAS2 cKO mice showed a reduced brain size (Fig. 3a, left) but not in the cerebellum, which agrees with the data in Fig. 2d showing equal BCAS2 expression between cKO and WT. The weight of the forebrain of cKO mice was 20% less than that of WT littermates (Fig. 3a, right), but the appearance and body weight was comparable to the WT (data not shown). Histology of the forebrain also revealed a smaller forebrain in cKO mice compared with WT mice (Fig. 3b). Hence, BCAS2 cKO in the forebrain resulted in a microcephaly-like phenotype.

Because the CaMKIIα-iCre transgene is widely expressed in projection neurons of the cerebral cortex, amygdala, hippocampus (including CA1, CA2, CA3 and CA4)^26^, the dentate gyrus (DG) is a critical entry point of the well-recognized hippocampal circuit, which conveys the neural signals from the performant pathway to the CA3 neurons that further project to CA1 neurons via the Schaffer collaterals. The activity of the hippocampal circuit is important for various cognitive functions and pathological conditions, and the circuit is gated at the main entrance, DG granule cells^19^. To evaluate the function of the hippocampus, we first examined the structural properties of DG granule cells in our mutant mice. To investigate the effect of BCAS2 elimination on DG volume, 40-μm vibratome sections from the hippocampus of adult WT and cKO mice were stained with anti-NeuN antibody (Fig. 3c). The DG volume evaluated using Fig. 3c was calculated by a stereologic method with ImageJ^29^,^30^. The DG volume of cKO mice was less than that of control mice (Fig. 3d; 0.40 vs 0.49 mm^3^; *P *< 0.05), which is consistent with the images in Fig. 3b,c. To further quantify the granule cell density in the DG, 4-μm coronal brain sections were incubated with anti-NeuN antibody (Fig. 3e). The granule cell density was greater in BCAS2 cKO than in WT mice (Fig. 3f, left. 2,788,235 vs 2,487,332 NeuN^+^ cells/mm^3^; *P *< 0.05). In addition, the estimated granule cell number was slightly decreased in cKO mice, but there was no significant difference in this number in the DG between WT and cKO mice (Fig. 3f, right). Therefore, the smaller DG volume in cKO mice may result from the reduced space within cells.

### BCAS2 cKO mice show impaired cognitive behaviors

**Figure 4:**
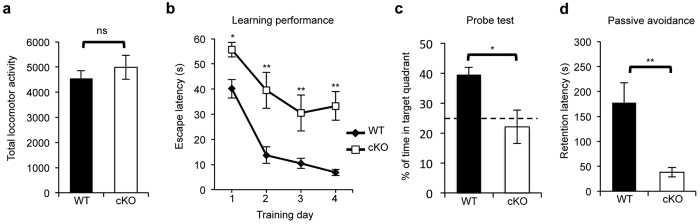
BCAS2 cKO mice show impaired cognitive behavior. (**a**) Locomotor activity in the open field test. cKO mice (n = 14) showed no difference compared to WT mice (n = 10) in the open field test analyzed by Student’s *t*-test. (**b**) Morris water maze. The latency of BCAS2 cKO mice to find the hidden platform was longer than that of WT for 4 constitutive training days. Two-way ANOVA (genotype versus day) revealed a major genotype effect and day effect for both groups. (**c**) Probe test after the Morris water maze, 24 hours after the last training day. The hidden platform was removed, and the percentage of time spent in the target quadrant was analyzed by Student’s *t*-test (WT, n = 5; cKO, n = 6). (**d**) Fear memory analysis. The passive avoidance test was performed using the Gemini avoidance system with a foot shock of 0.1 mA for 3 s. On the test day, the retention latency of the mouse in the light chamber was recorded, and the maximum time was set to 300 s. The data are shown as the mean ± S.E.M., and the *P* value was analyzed by a two-tailed Student’s *t*-test (WT: n = 5; cKO: n = 7). All tested mice of each genotype were at 12 weeks old.

Because BCAS2 cKO mice had small brains with significantly reduced DG volume (Fig. 3) and previous studies reported that brain size is associated with learning and memory capacities^31^,^32^, we evaluated the cognitive capabilities of BCAS2 cKO mice. In the open field test, the WT and cKO mice did not differ in locomotor activities, with the two genotypes having comparable mobility (Fig. 4a). To evaluate the hippocampus-dependent spatial learning and memory abilities, Morris water maze analysis was performed. On all 4 training days beginning with Day 1, BCAS2 cKO mice took a significantly longer time to find the platform than did WT. Thus, compared to WT mice, BCAS2 cKO mice showed reduced learning location ability (Fig. 4b). For the probe trial, the hidden platform was removed, and the memory consolidation after 4-day training trials was evaluated as the amount of time that the mice spent in the target quadrant (where the platform was placed). BCAS2 cKO mice showed a significant reduction in the retention latency in the target quadrant during the probe trial, which indicates impaired spatial memory (Fig. 4c). Because cKO showed impaired performances in learning and memory, we performed a passive avoidance test to evaluate the fear memory. The mice of each genotype were exposed to comparable fear-related learning and memory tasks. The apparatus consisted of one light and one dark compartment separated by a vertical sliding door. On the training day, the mouse entered the dark chamber and received an electrical foot shock. On the test day (24 hours after training), the time until the mouse entered the dark compartment was recorded as the retention latency. The latency time of the BCAS2 cKO in the light compartment was lower than that of WT (Fig. 4d), which indicates impaired fear memory consolidation in the cKO mice. Collectively, BCAS2 has a role in spatial and fear memory and learning ability.

### BCAS2 cKO in mice results in dendritic malformation

**Figure 5:**
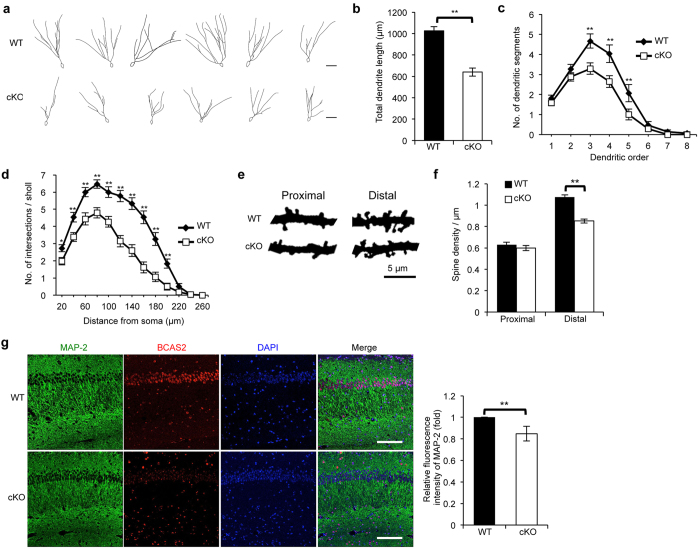
BCAS2 deletion causes reduced dendritic length. Golgi-impregnated brains were collected and analyzed from mice of each genotype at 12 weeks of age. (**a**) Golgi staining of the DG region of the hippocampus. Six examples of Golgi-stained DG granule neurons were reconstructed according to the Golgi impregnation method. Scale bar: 50 μm. (**b**) The dendritic length was analyzed using granule cells of each genotype (WT, n = 31 cells; cKO, n = 42 cells). The total dendritic length of cKO mice was significantly reduced. The data were analyzed using a two-tailed Student’s *t*-test analysis and are shown as the mean ± S.E.M. (**c**) Complexity of the dendritic arbor evaluated by plotting the number of dendritic segments against the dendritic order. Results are shown as the mean ± S.E.M. The *P* value was analyzed by two-way ANOVA (WT, n = 31 cells; cKO, n = 34 cells). (**d**) The dendritic complexity of BCAS2 cKO mice was less than that of WT, as judged by Sholl analysis. The number of intersections between dendrites and concentric rings was plotted against the distance from the soma. Two-way ANOVA revealed a significant difference for the genotype effect and distance effect for both groups (WT, n = 31 cells; cKO, n = 34 cells). (**e**) Dendritic spines of the proximal (<50 μm from soma) and distal (>100 μm from soma) regions in Golgi-stained DG granule cells. (**f**) Quantification of panel e. Proximal region: WT, n = 52 segments from 31 cells; cKO, n = 64 segments from 34 cells. Distal region: WT, n = 63 segments from 31 cells; cKO, n = 86 segments from 34 cells. Results are shown as the mean ± S.E.M. and analyzed by a two-tailed Student’s *t*-test. (**g**) IFA of the MAP-2 antibody (dendritic marker). Scale bar: 100 μm. Right: quantitation of the left panel (n = 3). Panels b, c, d and f were measured for each group with at least 5 independent experiments.

Brain neurons exhibit a complex dendritic distribution that plays a major role in forming neural circuits and process synaptic inputs^19^. To gain more insight into the defective hippocampus-related learning and memory in BCAS2 cKO mice, we used Golgi staining to examine the morphology of DG granule cells in the hippocampal region. We reconstructed 30 granule cells of each genotype for analysis (Fig. 5a) and found that the dendrite length was significant lower in cKO mice than in WT mice (Fig. 5b). Furthermore, we also examined the dendritic complexity of granule cells. The number of dendritic segments was significantly lower at each dendritic order in cKO mice compared with WT mice (Fig. 5c). Sholl analysis also revealed a lower number of intersections per Sholl in cKO mice compared with WT mice (Fig. 5d). Moreover, in BCAS2 cKO mice, the dendritic spine density of granule cells was significantly lower in the distal region but not in the proximal region of the DG (Fig. 5e,f). The association between BCAS2 and β-catenin in the mouse hippocampus, IFA revealed that cKO mice showed lower expression of β-catenin in the DG (Supplementary Fig. S3a,b). In BCAS2 cKO mice, the level of β-catenin was also decreased in the CA1 region (Supplementary Fig. S3c,d). Although we did not quantify the morphometric parameters of CA1 pyramidal neurons, immunostaining of microtubule-associated protein 2, a dendritic marker, showed impaired dendritic structures in CA1 neurons in BCAS2 cKO mice (Fig. 5g). It too indicated a β-catenin-mediated dendritic malformation in CA1 neurons of BCAS2 cKO mice. Collectively, BCAS2 is involved in dendritic growth.

### BCAS2 cKO mice have reduced dendrite growth in the newborn neurons of the adult DG

**Figure 6:**
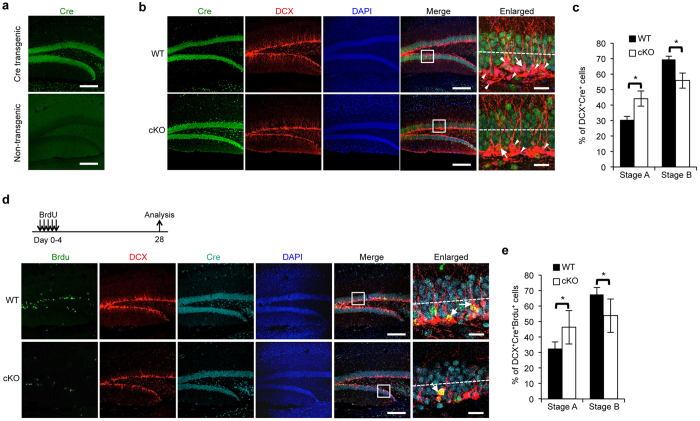
Lack of BCAS2 interrupts dendrite development in newborn neurons in DG. (**a**) IFA analysis of Cre expression. Sections of 40 μm from the DG of both CaMKIIα-iCre transgenic mice (upper) and non-transgenic mice lacking Cre gene (lower) were examined with anti-Cre antibody. Scale bar: 200 μm. (**b**) cKO mice show poor dendrite development in immature neurons. The 40 μm vibratome sections were immunostained with anti-DCX antibody and anti-Cre antibodies for each genotype at 6 weeks of age and counter-stained with DAPI (blue). White arrow: DCX^+^Cre^+^ immature neurons with longer primary dendrites in WT mice. White arrowhead: DCX^+^Cre^−^ immature neurons. Dotted line: line marking the middle of the granule cell layer (GL). Scale bars, 200 μm in Cre, DCX, DAPI and Merge panels; and 20 μm in enlarged panel. (**c**) Quantification of the percentage of each stage per 300 DCX^+^Cre^+^ cells from 3 mice of each genotype. Stage A: the axis of the newborn neurons is parallel to the GL or perpendicular to the GL with short processes. The length of the process should be less than half of the GL indicated by the dotted line. Stage B: newborn neurons with longer processes than neurons in stage A. The extended process should pass the middle of the GL and approach the molecular layer. (**d**) cKO mice show poor dendrite development in newborn neurons. Six-week-old mice of each genotype were injected with BrdU for five days and examined using IFA analysis of newborn neurons immunostained with anti-DCX, anti-Cre, anti-BrdU and counter-stained with DAPI (blue) on day 28 (upper). Representative images of DCX^+^Cre^+^BrdU^+^ triple positive cells by confocal microscopy (lower). Dotted line: line marking the middle of the GL. White arrow: DCX^+^Cre^+^BrdU^+^. Scale bars, 200 μm in Cre, DCX, DAPI and Merge panels; and 20 μm in enlarged panel. (**e**) Quantification of the percentage of each stage per 50 DCX^+^Cre^+^BrdU^+^ cells. The data are shown as the mean ± S.D. and analyzed by a two-tailed Student’s *t*-test.

DCX is an immature neuron marker, and nucleus BCAS2 was co-expressed with cytoskeleton DCX within one cell in normal mice (Supplementary Fig. S1c). We wondered whether BCAS2 could regulate dendritic development and growth of DG newborn neuron in Cre-expressing WT and cKO mice. Firstly, to test the specificity of the anti-Cre antibody, IFA was used and showed Cre expression in nucleus of CaMKIIα-iCre transgenic mice (WT); however, Cre was not detectable in the non-transgenic mice lacking Cre gene (Fig. 6a). Next we examined whether Cre (CamKIIα-driven) could be expressed in immature neurons. As shown in Fig. 6b (upper), DCX and Cre were co-expressed in the immature neurons of CaMKIIα-iCre transgenic mice and the proportion of Cre expression neurons was around 20% among total DCX-positive neurons that was in agreement with the report by Arruda-Carvalho *et al*.^33^. We then used IFA analysis to compare dendrite morphology of DCX^+^Cre^+^ using 40 μm sections for each genotype. To characterize dendrite morphology of DCX^+^Cre^+^ neurons, neurons that had a short dendrite that could not extend past half of the granule cell layer were considered stage A, and neurons that featured a long dendrite morphology (dendrites extended to half of the granule cell layer) were considered stage B^16^. DCX^+^Cre^+^ immature neurons in the DG of cKO mice had poor dendrite development compared with those of WT mice (Fig. 6b). Quantitation analysis revealed that in WT mice, 30.6% of DCX^+^Cre^+^ neurons were in stage A, and 69.4% were in stage B. In cKO mice, 44.2% of DCX^+^Cre^+^ neurons were in stage A, and 55.8% were in stage B (Fig. 6c). Moreover, to further examine the role of BCAS2 in the dendritic growth of newborn neurons, BrdU was administered, and IFA was performed after 28 days as previously described^16^ (Fig. 6d, upper panel). Co-expression of BrdU with DCX^+^Cre^+^ cells was found (Fig. 6d). In WT mice, 32.5% and 67.5% of BrdU^+^DCX^+^Cre^+^ cells belonged to stage A and B, respectively; compared with 46.3% and 53.7%, respectively, in cKO mice (Fig. 6e). Together, the results showed impaired dendritic growth in the immature neurons of BCAS2 cKO mice.

### Expression of β-catenin is reduced in BCAS2 cKO mice

**Figure 7:**
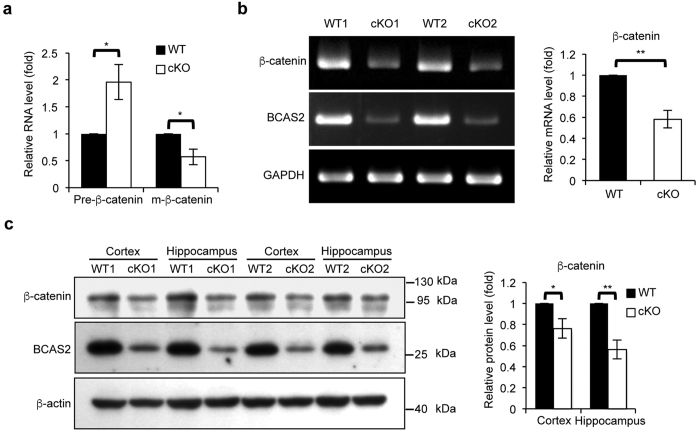
Reduced β-catenin gene expression in the BCAS2 cKO hippocampus. (**a**) RNA was extracted from the hippocampus of WT and BCAS2 cKO mice. Quantitative RT-PCR analysis showed that loss of BCAS2 caused increased β-catenin pre-mRNA and reduced β-catenin mRNA levels, as evaluated by three independent experiments, indicating the impaired splicing efficiency of β-catenin pre-mRNA (n = 3). (**b**) RT-PCR analysis of the β-catenin expression level. Left, representative result. Right, quantification of the left panel (n = 3). (**c**) Western blot analysis of the protein levels of BCAS2 and β-catenin in the cortex and hippocampus of each genotype. Left, representative western blot. Right, quantification of the left panel (n = 3). Results are shown as the mean ± S.D. and analyzed by a two-tailed Student’s test. Uncropped blots are presented in Supplementary Fig. S5.

As shown in Fig. 1, BCAS2 regulated β-catenin gene expression via pre-mRNA splicing. To further investigate the underlying mechanism of the abnormal cognitive behaviors and dendritic development of BCAS2 cKO mice; IFA revealed that cKO mice showed lower expression of β-catenin in the DG (Supplementary Fig. S3a,b). We then evaluated the splicing efficiency of β-catenin by BCAS2 in cKO mice, RNAs from the hippocampal tissues of each genotype were analyzed. The results showed an accumulation of pre-RNA and a reduced mRNA level in BCAS2 cKO mice compared with WT (Fig. 7a). Moreover, RT-PCR and western blot analysis further confirmed the decreased β-catenin expression level in the forebrain of BCAS2 cKO mice compared with WT (Fig. 7b,c). Thus, BCAS2 regulates the β-catenin pre-mRNA splicing in the forebrain, which is consistent with the data shown in Fig. 1.

Furthermore, we previously reported that *Drosophila* BCAS2 participates in *Delta* pre-mRNA splicing^12^. Therefore, we also examined whether BCAS2 elimination resulted in impaired splicing efficiency of *Delta* pre-mRNA in mammalian cells. A splicing assay in MCF7 cells revealed that the *Delta* gene splicing was indeed regulated by BCAS2 (Supplementary Fig. S4a), in agreement with our previous report^12^. However, in the hippocampal tissues of cKO mice, the *Delta* mRNA and protein expression did not differ between WT and cKO mice (Supplementary Fig. S4b,c). Therefore, the expression of Delta is not regulated by BCAS2 in the hippocampus of mice, and BCAS2-regulated dendritic development is independent of Delta-Notch signaling. Altogether, the results indicate that BCAS2 may regulate dendrite formation and growth via β-catenin.

### Overexpression of β-catenin can rescue dendritic growth in BCAS2-knockdown primary neurons

**Figure 8:**
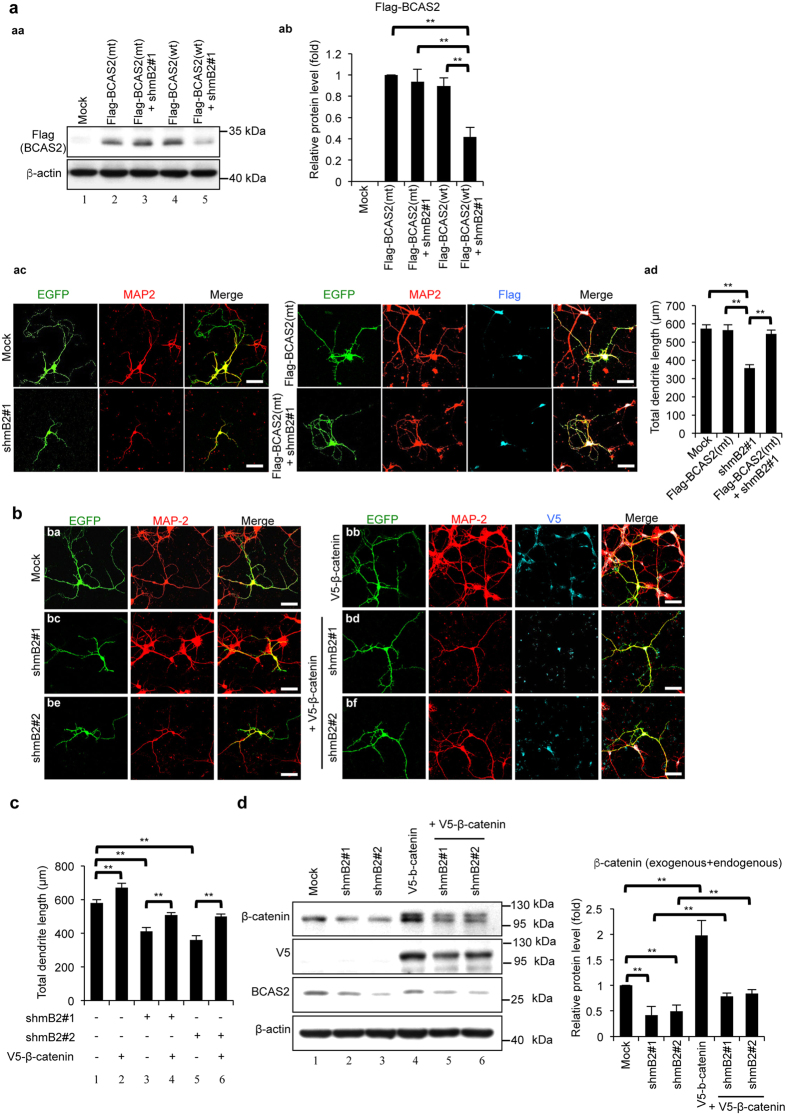
Overexpression of β-catenin can rescue the impaired dendritic growth of the BCAS2 knockdown in a primary neuron culture. (**a**) Identification of shBCAS2 to target mouse BCAS2 gene. (aa) Western blot analysis. shmB2#1 could not reduced the exogenous BCAS2 expression while co-transfected with BCAS2 silent mutant (Flag-BCAS2(mt)). (ab) Quantification of three independent experiments analyzed by one-way ANOVA. (ac) BCAS2 silent mutant can rescue dendrite length in shRNA treated primary neuron cells. The primary neurons isolated from the forebrains of E18.5 embryos were seeded and then transfected with the following: the pLL3.7 (Mock), shRNAs targeting BCAS2 (shmB2#1) or Flag-tagged BCAS2(mt) alone, or shBCAS2 plasmid with Flag-tagged BCAS2(mt). IFA was performed 5 days after the plasmid transfection using anti-MAP-2 and anti-Flag antibodies. Dendritic length was measured from 30 merged EGFP/MAP-2 double positive (ac, left panels) and EGFP/MAP-2/Flag triple positive cells (ac, right). (ad) Quantification of dendrite length. (**b**) β-Catenin can restore the dendrite length of BCAS2-depleted primary neurons. Scale bar: 20 μm. The primary neurons were transfected with the following: the pLL3.7 backbone EGFP-containing vector (Mock) (panel ba), shRNAs targeting BCAS2 (shmB2#1 or shmB2#2) (panels bc and be) or V5-tagged β-catenin alone (panel bb), or shBCAS2 plasmids with exogenous V5-tagged β-catenin plasmids (panels bd, and bf). IFA was performed 5 days after the plasmid transfection using anti-MAP-2, anti-V5 antibodies (indicating exogenous β-catenin). Dendritic length was measured from 50 merged EGFP/MAP-2 double positive (panels ba, bc, and be) and EGFP/MAP-2/V5 triple positive cells (panels bb, bd, and bf). (**c**) Quantification of dendrite length measured from 50 EGFP-positive neurites of each group. The *P* value was analyzed by one-way ANOVA. The data are shown as the mean ± S.E.M. (**d**) Western blot analysis of BCAS2, V5 (exogenous β-catenin) or β-catenin (endogenous) in N2A cells transiently transfected with the plasmids described in panel b. Left: representative western blot. Right: quantification analysis. The results are shown as the mean ± S.D. and analyzed by one-way ANOVA (n = 3). Uncropped blots are presented in Supplementary Fig. S5.

Dendritic spines are small actin-rich protrusions from neuronal dendrites that contribute to the postsynaptic information processing of most excitatory synapses associated with learning and memory^34^. BCAS2 cKO mice showed abnormal cognitive behavior, dendritic malformation and a low level of β-catenin expression in the forebrain. Here, to further confirm that BCAS2 regulates dendrite growth through β-catenin *in vivo*, we induced β-catenin expression in BCAS2-knockdown primary neurons. The shBCAS2 (shmB2#1) targeting the mouse BCAS2 gene was then generated, and it could efficiently diminish exogenous BCAS2 expression in N2A cells in which containing Flag-BCAS2(wt) (Fig. 8aa, lanes 4 and 5). To further test the specificity of shmB2#1, we generated BCAS2 mutant plasmid for a silent mutant resistant to shmB2#1; the sequence corresponding to the shmB2#1-targeted nucleotide sequence (position 277–293) and was named Flag-BCAS2(mt). When Flag-BCAS2(mt) and shmB2#1 were co-transfected into N2A cells, the results showed that Flag-BCAS2(mt) could express BCAS2 protein as Flag-BCAS2(wt) (Fig. 8aa, lanes 2 and 4) and shmB2#1 could not reduce exogenous BCAS2 expression in Flag-BCAS2(mt)-containing cells (Fig. 8aa, lane 3). Quantitation analysis showed in Fig. 8ab. Additionally, BCAS2 silent mutant could rescue the reduced dendrite length in BCAS2-knockdown primary neurons those were isolated from the forebrains of E18.5 embryos (Fig. 8ac,ad). Collectively, shBCAS2 can specifically inhibit BCAS2 expression. To test whether β-catenin can rescue dendritic growth in BCAS2-knockdown primary neurons, we transfected the neurons with enhanced green fluorescent protein (EGFP)-containing shBCAS2 plasmid alone or with exogenous V5-tagged β-catenin plasmids. We measured the dendrite length using the existence of MAP2 along with the overlapping GFP and V5 expression in the neuron. As shown in Fig. 8b and with the quantitative analysis in Fig. 8c, the overexpression of β-catenin alone significantly increased the dendrite growth compared with that of the control (Fig. 8b, panel bb; Fig. 8c, lane 2). For the BCAS2 knockdown, reduced dendrite growth was observed (Fig. 8b panels bc and be; Fig. 8c, lanes 3 and 5). However, forced β-catenin expression in BCAS2-knockdown cells restored the decreased dendrite growth (Fig. 8b panels bd and bf; Fig. 8c, lanes 4 and 6). Collectively, the results indicate that β-catenin could restore the dendrite length in BCAS2-knockdown primary neurons. Moreover, each shRNA targeted to BCAS2 decreased the level of BCAS2 and decreased the endogenous β-catenin level (Fig. 8d, lanes 2 and 3), in agreement with Fig. 7c. The exogenous V5-tagged β-catenin was detectable in BCAS2 knockdown cells and restored the decreased β-catenin expression (Fig. 8d, lanes 4–6). In summary, BCAS2 regulates neuron growth in a β-catenin-dependent manner.

## Discussion

Here, we demonstrated that BCAS2 regulates β-catenin pre-RNA splicing. An exon array assay indicated that β-catenin was a candidate of BCAS2 splicing-target gene (Supplementary Table S1). We confirmed that a lack of BCAS2 reduced the β-catenin pre-mRNA splicing, leading to reduced β-catenin expression in the hippocampal tissues of BCAS2 cKO mice (Fig. 7). Hence, BCAS2 is an upstream regulator of β-catenin gene expression. A lack of β-catenin leads to early embryonic death^35^. β-Catenin is highly expressed in the entire brain, especially the cortex and hippocampus, and it plays a critical role in dendritic arborization and dendritic complexity, including the dendritic branch tip number and the total dendritic branch length, during post-natal neurogenesis^16^,^18^,^24^. Loss of function or mutations at individual residues of β-catenin causes brain malformation^36^,^37^. β-catenin cKO in postnatal newborn neurons in the hippocampal DG leads to defective dendritic morphology^16^. Like β-catenin, BCAS2 is highly expressed in the brain (Supplementary Fig. S1), and global BCAS2 KO is embryonically lethal^10^. These findings imply a strong functional correlation between BCAS2 and β-catenin.

BCAS2 can regulate the expression level of β-catenin, which reportedly regulates dendrite growth^16^,^18^. In this study, BCAS2 cKO mice showed decreased dendrite elongation during dendritic development in the postnatal DG. Overexpression of β-catenin with BCAS2 knockdown in primary neurons restored dendritic growth (Fig. 8). These results are consistent with the increased dendritic arborization when β-catenin is expressed exogenously^18^. Thus, BCAS2 cKO mice exhibit dendritic malformation, at least partially, via reduced β-catenin expression.

Here, we showed that BCAS2 knockout in the forebrain led to a reduced brain size and impaired learning and memory. In terms of brain morphology, BCAS2 cKO mice exhibited a microcephaly-like phenotype (Fig. 3). The reduced forebrain size may result from several factors, such as a lack of newborn neurons, cell death, or changes in dendrite length and complexity. However, the NeuN-positive cells in the DG were not significantly different between WT and BCAS2 cKO mice, and the NeuN-positive cell density in BCAS2 cKO mice was greater than that of WT (Fig. 3). It is clear that β-catenin, a downstream effector of BCAS2, is expressed in hippocampal neurons^16^. In the present study, IFA demonstrated the reduced level of β-catenin in the hippocampus of BCAS2 cKO mice (Fig. S3). In cKO mice, shortened dendritic length and reduced dendritic complexity were evident in DG granule cells and DCX-positive immature neurons revealed by Golgi-impregnation (Fig. 5) and immunostaining (Fig. 6), respectively. Since β-catenin is known to play important roles in dendritic development in the hippocampal DG. Our results are in line with these findings; hence β-catenin may mediate the effect of BCAS2 on dendritic arbors of DG neurons.

BCAS2 cKO mice showed learning and memory defects in both the Morris water maze assay and the passive avoidance test. A small brain size is strongly associated with cognitive deficits. For example, chemical-induced rat microcephaly produces neurobehavioral abnormalities associated with congenital brain defects^31^. Patients with decreased extracellular signal-regulated kinase 2 (ERK2) protein levels show clinical microcephaly with learning disabilities^38^, and ERK2-null mice show microcephaly associated with impaired proliferation of neural progenitors^32^. Moreover, aging can trigger mild cognitive impairment and dementia that can be diagnosed by hippocampal atrophy^39^,^40^. In contrast, a large hippocampus is closely associated with good memory and cognitive function^40^,^41^. It is worthwhile to investigate the volume of various regions in the forebrain (such as the cerebral cortex) and the progenitor proliferation of BCAS2 cKO to obtain more insight into the BCAS2-null-induced microcephaly.

In general, neurogenesis involves neural stem cell (NSC) proliferation and differentiation, axonal and dendritic specification, NSC maturation, and the synaptogenesis of newborn cells. Here, we used BrdU tracing showed that the loss of BCAS2 would impair the dendritic growth of newborn neurons (Fig. 6d,e). Thus, the impaired dendritic development in cKO mice further suggests that a lack of BCAS2 may cause neurodegenerative diseases, which supports previous reports of Alzheimer’s disease (AD) patients showing low mRNA expression of BCAS2^42^. Moreover, AD patients also show a reduced hippocampal volume^43^. Whether and how BCAS2 is involved in AD needs further investigation. Furthermore, neurodegenerative diseases are induced by a gradual loss of different neuron populations, and they often feature axonal and dendritic degeneration, which leads to synaptic transmission deficits^44^,^45^. Apart from AD, several neuronal disorders, such as schizophrenia^46^, major depression^47^ and temporal lobe epilepsy^48^, show reduced hippocampal volume^49^. The shrinkage of the hippocampus in late adulthood leads to impaired memory and increased risk for dementia^50^. Here, we found that BCAS2 cKO mice had a small brain size with impaired learning and memory, and they also exhibited markedly defective dendritic arborization of the hippocampus (Figs 3, 4, 5 and 6). These findings are consistent with impaired dendritic development leading to hippocampal atrophy with effects on memory^51^.

BCAS2 regulates RNA splicing^7^,^8^. *Drosophila* BCAS2 reportedly participates in *Delta* pre-mRNA splicing and regulates Delta-Notch signaling in *Drosophila* wing development^12^. In both the neurogenic SGZ and subventricular zone (SVZ) regions, Delta-Notch signaling maintains the population of neuron stem cells^52^ and is also involved in dendritic development in the hippocampal DG^52^. In MCF7 cells, BCAS2 regulates *Delta* RNA splicing (Supplementary Fig. S4a), as previously reported^12^. However, the mRNA and protein expression of *Delta* did not differ in the brain tissues of WT and BCAS2 cKO mice (Supplementary Fig. S4b,c). Thus, BCAS2 may not regulate the dendritic development in the hippocampal region through Delta-Notch signaling. Similarly, the splicing of multiple tested RNAs, including critical cell cycle regulators, such as p53, cyclin D1, cyclin E1, and tubulin, in PLRG1-deficient mouse embryo fibroblasts was unaffected^21^. Hence, various tissues may assemble different splicing factors for specific gene splicing to control the gene expression pattern.

Microarray analysis of AD patients revealed decreased expression of BCAS2 and β-catenin in; thus, these genes are categorized as AD-related genes^42^. A single nucleotide polymorphism in the BCAS2 gene loci is associated with autism^53^. Children with autism can have mental disorders. Thus, decreased BCAS2 expression or a BCAS2 mutation may be involved in neuronal degenerative diseases. Current therapeutic approaches to elevate the β-catenin level (such as GSK-3β inhibitor) in neurodegenerative diseases are in development^54^. We found that BCAS2 could regulate the β-catenin level, which can regulate dendrite growth. Hence, BCAS2 may also be a therapeutic target for neurodegenerative diseases.

In summary, BCAS2 is an upstream regulator of β-catenin gene expression, and it plays a role in dendrite growth, at least partially through β-catenin. Our data suggest that the BCAS2-null-induced small brain size may result from dendrite malformation, thus resulting in cognitive deficits.

## Materials and Methods

### Exon array analysis

RNA was extracted with TRIzol reagent and then purified with an RNeasy Minikit (Qiagen) from MCF7 after depleting BCAS2 by shBCAS2#14. RNA samples were analyzed by the microarray core facility at the National Health Research Institute (NHRI, Taiwan) using an Affymetrix Human Exon 1.0 ST array. The alternative splicing events and gene expression profiles were predicted and generated by AltAnalyze software according to the online manual (https://code.google.com/p/altanalyze/wiki/AboutUs#Citing).

### Construction of a targeting vector and BCAS2-floxed mice

A 16.1-kb fragment of mouse genomic DNA containing exons 1 to 7 of the BCAS2 gene was retrieved from the BAC clone (bMQ-122M7). The fragment was inserted into the NotI-SpeI site of the pL253 vector, in which MC1-TK (thymidine kinase) provides a negative selection marker^55^. This construct was then used as a backbone for inserting a LoxP sequence from pL452 and a Neo cassette (5′-FRT-PGK-NeobpA-FRT-loxP-3′) from pL451 into introns 2 and 4, respectively. The final targeting construct contained 8.1 kb homologous arms on the 5′ end and 4 kb arms on the 3′ end. The targeting vector was linearized by DNA digestion at the unique NotI site and electroporated into R1 hybrid ES cells. Targeted clones were selected by G418. The G418-resistant ES cells were injected into blastocytes of C57BL/6JNarl origin and implanted into pseudo-pregnant foster mothers after candidate ES cells were verified by Southern blotting for the homologous recombination event (data not shown). The resulting chimeras were mated to establish germ-line transmission, and heterozygous (BCAS2^Flox/+^) animals were intercrossed to establish the BCAS2^Flox/Flox^ line of mice.

### Generation and genotyping of BCAS2 conditional knockout mice

To obtain forebrain-specific knockout mice, BCAS2^Flox/Flox^ mice were crossed with CaMKIIα-iCre transgenic mice to obtain BCAS2^Flox/+^;CaMKIIα-iCre^+^ mice. The CaMKIIα-iCre transgenic mice expressing Cre recombinase was regulated by the CaMKIIα promoter in a BAC-expressing vector^26^. The presence of the Cre gene was verified by PCR. Then, BCAS2^Flox/+^;CaMKIIα-iCre^+^ offspring were crossed with each other, resulting in six different genotypes (Fig. 2b). Only BCAS2^Flox/Flox^;CaMKIIα-iCre^+^ mice were considered forebrain-specific BCAS2 cKO mice and were named cKO in this study; the WT mice were the BCAS2^+/+^ CaMKIIα-iCre^+^ transgenic mice that contained the Cre gene. To confirm the genotypes of the offspring, genomic DNA was extracted from the mouse tail using DirectPCR Lysis Reagent (Viagen) and analyzed by PCR.

### Immunohistochemistry (IHC) and immunofluorescence assay (IFA)

Mice were anesthetized with avertin, transcardially perfused with 4% paraformaldehyde (PFA), and then sectioned coronally at 40 μm by a vibratome for free-floating sections^30^. The paraffin-embedded tissues were sectioned at either 4 or 10 μm. For Nissl staining, 4-μm brain sections were stained with 1% cresyl violet (Sigma-Aldrich). For IHC and IFA, paraffin-embedded sections or free-floating sections were incubated with the indicated antibodies (Supplementary Table S2). For IFA, fluorescence signals were produced by incubation with Cy3-conjugated goat anti-mouse or Alexa Fluor 488-conjugated goat anti-rabbit secondary antibody. The images were acquired with a Leica TSC SP5 confocal microscope.

### Golgi staining

Brain samples were incubated in the impregnation solution of a Rapid GolgiStain kit (FD NeuroTecnologies) at room temperature for 3 weeks. Samples were sliced into 150-μm sections using a vibratome. Morphological analysis of DG granule cells involved light microscopy (Olympus) combined with Stereo Investigator system software (Microbrightfield Bioscience). The granule cell morphology was reconstructed, Scholl analysis was performed, and the dendritic length, dendritic segments, and branch order were analyzed using Neurolucida and Neurolucida Explorer software (Microbrightfield Bioscience). The density of dendritic spines was determined using ImageJ software (US National Institutes of Health).

### DG volume analysis

A series of every 12th vibratome section (480 μm apart) throughout the hippocampus was examined using IHC with anti-NeuN antibody and was digitized with a 10× objective lens^29^. The DG area of every section was measured using ImageJ. The DG volume was calculated by multiplying the DG area with the section thickness (40 μm) and the number of sections (1-in-12 series of coronal sections) to obtain DG volume = (sum of the NeuN^+^ DG areas in every section) × 40 × 12.

### Dendrite growth

For detection of the dendrite growth, mice of each genotype were intraperitoneally injected with BrdU at 50 mg/kg daily for 5 consecutive days and then scarified 28 days after the first BrdU injection. Fixative brains were prepared as serial vibratome sections for IFA analysis. Series of every 12^th^ vibratome section throughout hippocampus were first rinsed in PBS for 5 min, incubated in 2N HCl at 40 °C 30 min followed by 0.1 M borate buffer (pH 8.4) at room temperature for 10 min, and then followed the IFA procedure. The morphological characteristics of DCX^+^Cre^+^ and BrdU^+^DCX^+^Cre^+^ neurons were divided into two categories; stage A of immature neurons was defined as a lack of dendrites or the presence of only short dendrites, and stage B featured a long and complicated dendrite morphology^16^.

### Behavioral tests

Mice at 8 weeks of age were habituated to the test environments for 30 min before all behavioral tests^56^. The locomotor activity was evaluated in open field boxes (40 × 40 cm; San Diego Instruments). For the Morris water maze, a swimming pool (100 cm in diameter) was divided into four quadrants. Every mouse underwent 4 training trials per day (15-min interval between trails) for 4 consecutive training days. In each trial, the mice started swimming from the diagonal quadrant of the target quadrant (where the platform located) and were then allowed to explore the platform hidden beneath the water surface for 60 s. The duration of exploration was measured from the initial swimming to standing on the platform and defined as the escape latency. Twenty-four hours after the last training trial, the platform was removed, and all mice were given a one-minute probe trial to test whether they remembered the location of the platform (target quadrant). The mice were tracked using EthoVision 3 (Noldus Information Technology Inc.). For passive avoidance, mice were examined with the Gemini avoidance system (San Diego Instruments) as previously described^56^ with a modification in which the foot shock condition was set to 0.1 mA/10 g body weight for 3 s. Briefly, on the first day, the mouse was placed in the light compartment and allowed to explore the environment for 30 s. After exploration, the guillotine door between the light and dark compartments was raised, and the time used by each mouse to enter the light compartment was recorded. On the second day, once the mouse entered the dark compartment with all four paws, an electric shock was administered, and then the mouse was returned to the home cage. On the third day, the mouse was placed in the light chamber again. After the guillotine door was raised, the retention time of the mouse in the light chamber was recorded, and the maximum time was set to 300 s.

### Western blot analysis

Protein lysates were extracted and subjected to western blot analysis with the antibodies indicated in Supplementary Table S2 according to a previously described method^4^.

### RT-PCR and quantitative PCR

The total RNA of cells and murine forebrains was extracted using TRI reagent (Invitrogen). Oligo-dT and random hexamers were used to synthesize cDNA for detecting mRNA and pre-mRNA, respectively. The sequences of primers are listed in Supplementary Table S3.

### Cell culture and transfection

Plasmids and siRNA were transiently transfected into MCF7 and N2A cells for 48 h using jetPRIME (Polyplus). The method of isolating primary neurons from forebrains of E18.5 embryos has been previously described^57^. Briefly, coverslips were precoated with 1 mg/ml poly-D-lysine; then, primary neurons were seeded at 10^5^ cells/well in 24-well plates. Cells were cultured in Neurobasal medium (Invitrogen) supplemented with 2% B27 (Invitrogen), 0.5 mM L-glutamine, 100 U/ml penicillin, and 100 mg/ml streptomycin. Transfection was performed using the calcium phosphate method. After transfection for 5 days, cells were fixed and analyzed using immunofluorescence, and the dendrite length was measured. The dendrites of primary neurons were measured using the MAP-2 expression in protrusions >10 μm from the soma; then, calculations were performed using Neurolucida and Neurolucida Explorer software (Microbrightfield Bioscience). The fluorescence images of primary neurons were obtained by confocal microscopy (Leica TCS SP5).

### Statistical analysis

Statistical comparisons were performed using the two-tailed Student’s *t*-test, one-way ANOVA, and repeated measure two-way ANOVA (followed by *post hoc* Bonferroni’s test) for independent samples with Microsoft Excel 2010 and StatPlus software. The data are presented as the mean ± SEM or SD. *P < 0.05 and **P < 0.01 were considered statistically significant.

### Study approval

All procedures of the animal experiments were reviewed and approved by the Institutional Animal Care and Use Committee at the College of Medicine, National Taiwan University (IACUC) and all experiments were performed in accordance with the approved relevant guidelines and regulations. All experimental mice were housed in the animal center under a 12-h light/dark cycle with free access to food and water.

## Additional Information

**How to cite this article**: Huang, C.-W. *et al*. Conditional Knockout of Breast Carcinoma Amplified Sequence 2 (BCAS2) in Mouse Forebrain Causes Dendritic Malformation via β-catenin. *Sci. Rep.* **6**, 34927; doi: 10.1038/srep34927 (2016).

## Supplementary Material

Supplementary Information
