# Supplementary material for: Conditional Knockout of Breast Carcinoma Amplified Sequence 2 (BCAS2) in Mouse Forebrain Causes Dendritic Malformation via β-catenin
Source: Sci Rep. 2016 Oct 7;6:34927. doi: 10.1038/srep34927 (PMC5054673; doi:10.1038/srep34927)
Supplement: Supplementary Information [file srep34927-s1.pdf]

Supplementary Information

Conditional Knockout of Breast Carcinoma Amplified Sequence 2 (BCAS2) in Mouse Forebrain Causes Dendritic Malformation via β-catenin

Chu-Wei Huang[1], Yi-Wen Chen[1], Yi-Rou Lin[1], Po-Han Chen[1], Meng-Hsuan Chou[1], Li-Jen Lee[2], Pei-Yu Wang[3], June-Tai Wu[4,5], Yeou-Ping Tsao[6], Show-Li Chen[1*]

Gene-Set Name	No. changed	% changed	Z Score	gene symbols
mRNA processing:WP411	60	48.7804878	5.313390142	CDC40, CELF1, CLASRP, CLK1, CLP1, CPSF2, CPSF3, CSTF2T, CSTF3, DDX1, DDX20, DHX9, DNAJC8, EFTUD2, HNRNPA2B1, HNRNPA3, HNRNPAB, HNRNPD, HNRNPH1, HNRN HNRNPU, METTL3, NCBP1, NCBP2, PAPOLA, POLR2A, PPM1G, PRMT2, PRPF3, PRPF40A, PRPF4B, PRPF8, PTBP2, RBM17, RBM39, RBM5, RBMX, RNMT, SF3A1, SF3A3, SFSWAP, SMC1A, SNRPA1, SNRPD1, SNRPD3, SPOP, SREK1, SRPK1, SRPK2, SRRM1, SRSF1, SRSF10, SRSF3, SRSF5, SRSF7, SRSF9, SUGP1, SUGP2, TRA2B, XRN2
G1 to S cell cycle control:WP45	35	51.47058824	4.41994258	ATM, CCNB1, CCNE1, CCNE2, CCNG2, CDC25A, CDK1, CDK2, CDK6, CDK7, CREB3L4, E2F3, E2F6, MCM2, MCM4, MCM6, MDM2, MNAT1, MYT1, ORC2, ORC3, ORC4, ORC5, ORC6, POLA2, POLE, POLE2, PRIM1, PRIM2, RB1, RBL1, RPA3, TFDP1, TFDP2, WEE1
DNA Replication:WP466	24	57.14285714	4.286960523	CDC6, CDC7, CDK2, CDT1, GMNN, MCM2, MCM4, MCM6, ORC2, ORC3, ORC4, ORC5, ORC6, POLA1, POLA2, POLD3, POLE, POLE2, PRIM1, PRIM2, RFC1, RFC3, RPA3, UBA5
p38 MAPK Signaling Pathway:WP400	19	61.29032258	4.195451588	ATF2, CDC42, MAP2K4, MAP2K6, MAP3K1, MAP3K5, MAP3K7, MAPK14, MAPKAPK5, MAX, MEF2D, MKNK1, RASGRF1, RIPK1, RPS6KA5, STAT1, TGFB2, TGFBR1, TRAF2
Eukaryotic Transcription Initiation:WP405	22	55	3.877979659	CDK7, ERCC3, GTF2A2, GTF2E1, GTF2H2, GTF2H3, MNAT1, POLR1A, POLR1B, POLR1D, POLR1E, POLR2A, POLR2B, POLR2K, POLR3B, POLR3D, POLR3E, TAF12, TAF5, TAF6, TAF9, TBP
Cell cycle:WP179	45	44.55445545	3.837012751	ATM, ATR, BUB1, BUB1B, BUB3, CCNA2, CCNB1, CCNE1, CCNE2, CDC14A, CDC14B, CDC25A, CDC25B, CDC25C, CDC6, CDC7, CDK1, CDK2, CDK6, CHEK2, E2F3, E2F6, HDAC1 HDAC2, HDAC4, HDAC7, MCM2, MCM4, MCM6, MDM2, ORC2, ORC3, ORC4, ORC5, ORC6, PRKDC, RB1, RBL1, SMAD4, SMC1A, TBC1D8, TFDP1, WEE1, YWHAB, YWHAE
EGF/EGFR Signaling Pathway:WP437	57	41.60583942	3.705918962	AP2A1, AP2B1, ATXN2, BRAF, CAV1, CDC42, EGF, EGFR, ELK4, EPS15, EPS8, ERBB2, GAB2, HGS, ITCH, JAK1, JAK2, JUN, KRAS, LIMK2, MAP2K5, MAP3K1, MAP3K2, MAP3K4 MAPK14, MAPK8, MAPK9, PIAS3, PIK3C2B, PIK3R1, PLSCR1, PRKCI, PRKCZ, PTK2, PTK2B, PTPN12, RAF1, RALA, RALB, RALBP1, RALGDS, RAP1A, RASA1, REPS2, RICTOR, RPS6KA5, SH3GL2, SH3KBP1, SOS1, SOS2, STAM, STAM2, STAMBP, STAT1, STMN1, STXBP1, VAV3
TNF alpha Signaling Pathway:WP231	38	44.70588235	3.543479102	BIRC2, CFLAR, CREBBP, CSNK2A1, CUL1, CYBA, DIABLO, FBXW11, GLUL, GLULP4, IKBKB, JUN, MADD, MAP2K4, MAP2K6, MAP3K1, MAP3K5, MAP3K7, MAP4K2, MAPK8, MAPK9, NFKB1, PPP2CA, PRKCZ, PSMD2, PYGL, RAF1, REL, RFK, RIPK1, SKP1, SMPD2, SOS1, TAB2, TAB3, TANK, TBK1, TRAF2
DNA damage response:WP707	32	45.07042254	3.301377248	ATM, ATR, ATRIP, BRCA1, CCNB1, CCNE1, CCNE2, CDC25A, CDC25C, CDK1, CDK2, CDK5, CDK6, CHEK2, CYCS, FANCD2, MDM2, NBN, NPAT, PIDD, PRKDC, RAD1, RAD52, RAD9A, RB1, RFC1, RRM2B, SESN1, SMC1A, TLK1, TLK2, TREX1
MAPK signaling pathway:WP382	61	38.85350318	3.190854313	AC013461.1, ACVR1B, AKT2, ARRB1, ATF2, BRAF, CASP2, CASP6, CDC25B, CDC42, DUSP10, EGF, EGFR, IKBKB, JUN, KRAS, LAMTOR3, MAP2K4, MAP2K5, MAP2K6, MAP3K1 MAP3K13, MAP3K4, MAP3K5, MAP3K6, MAP3K7, MAP4K3, MAP4K4, MAPK10, MAPK14, MAPK8, MAPK9, MAPKAPK5, MAPT, MAX, MRAS, NF1, NFKB1, PAK2, PPM1B, PPP3CB, PPP3CC, PPP3R1, PRKCH, PRKCZ, PTPRR, RAF1, RAP1A, RASA1, RASA2, RASGRF2, RASGRP1, SOS2, STK3, STMN1, TAB2, TGFB2, TGFBR1, TMEM37, TRAF2, TRAF6
Insulin Signaling:WP481	61	38.36477987	3.071210199	AKT2, ARHGAP33, CAP1, CBLB, CYTH3, ENPP1, FLOT2, GRB10, GRB14, IGF1R, IKBKB, INPP4A, INSR, IRS1, JUN, MAP2K4, MAP2K5, MAP2K6, MAP3K1, MAP3K13, MAP3K2, MAP3K4, MAP3K5, MAP3K6, MAP3K7, MAP4K2, MAP4K3, MAP4K4, MAP4K5, MAPK10, MAPK14, MAPK8, MAPK9, MTOR, PIK3CA, PIK3CB, PIK3R1, PIK3R3, PIK3R4, PRKA PRKCH, PRKCI, PRKCZ, RAB4A, RAF1, RAPGEF1, RHOQ, RPS6KA4, RPS6KA5, SGK1, SGK3, SLC2A1, SNAP23, SNAP25, SOS1, SOS2, STXBP1, STXBP3, STXBP4, TSC1, TSC2
TCA Cycle:WP78	17	51.51515152	3.069553505	CS, DLD, DLST, FH, IDH3A, IDH3B, ITGA6, MDH2, OGDH, PDHB, PDK1, PDK4, PDP1, RP4-682C21.2, SDHA, SDHD, SUCLG2
TGF beta Signaling Pathway:WP366	46	40	2.993468427	ATF2, CAV1, CDC42, CDK1, COPS5, CUL1, DCP1A, ETS1, FOXP3, HDAC1, HGS, ITCH, ITGA6, JUN, LIMK2, MAP2K4, MAP2K6, MAP3K7, MAPK14, MAPK8, MAPK9, MEF2A, NUP214, PAK2, PDK1, PIK3R1, PTK2, RAF1, RBL1, RNF111, SKIL, SKP1, SMAD4, SNIP1, SOS1, SPTBN1, SUMO1, TERT, TFDP1, TGFBR1, TGIF1, TRAF6, UCHL5, WWP1, ZFYVE1 ZFYVE9
Fatty Acid Biosynthesis:WP357	12	54.54545455	2.820910874	ACAA2, ACACB, ACSL1, ACSL3, ACSL6, DECR1, ECH1, ECHDC1, ECHDC2, MECR, PECR, SCD
Androgen receptor signaling pathway:WP138	34	41.46341463	2.815274022	BAG1, BRCA1, CAV1, CCNE1, CDC42, CREBBP, CTNNB1, DSTN, EGFR, GNB2L1, HDAC1, JUN, KAT2B, KDM1A, LIMK2, MDM2, NCOA1, PARK7, PIAS3, PIK3R1, PPAP2A, PTK2, RAD9A, RAN, RB1, RNF14, RNF4, ROCK1, ROCK2, SMAD4, SMARCE1, SUMO1, UBE3A, ZMIZ1
FAS pathway and Stress induction of HSP regulation:WP314	17	47.22222222	2.629045898	CASP6, CFLAR, CYCS, DFFA, FAF1, JUN, LMNB1, MAP2K4, MAP3K1, MAP3K7, MAPK8, MAPKAPK3, PAK2, PARP1, PRKDC, RB1, RIPK2
TSH signaling pathway:WP2032	27	42.1875	2.611358818	APEX1, BRAF, CCNE1, CDK2, GNA13, GNAI2, GNAI3, GNAQ, GNB1, IGF1R, JAK1, JAK2, JUN, KCNIP3, MAP2K6, MAPK14, MTOR, PAX8, PDE4D, PIK3CA, PIK3R1, RAF1, RALGD RAP1A, RB1, STAT1, TTF1
DNA damage response (only ATM dependent):WP710	34	40	2.562474079	APC, ATM, BCL2L11, BCL6, CCNG2, CDC42, CTNNB1, DVL2, ERBB2, HMGB1, INSR, IRS1, ITGA6, JUN, LDLR, MAP3K1, MAP3K4, MAP3K7, MAPK10, MAPK8, MAPK9, MDM2, MLKL, NPAT, PCK2, PDK1, PPP2R5C, PPP2R5E, RAC1, SCP2, SMAD4, SOS1, SOS2, WNT2B
B Cell Receptor Signaling Pathway:WP23	36	39.13043478	2.479800873	ATF2, BCL6, BLNK, BRAF, CARD11, CD22, CDC42, DAPP1, E2F3, ETS1, GAB2, GTF2I, IKBKB, ILF2, IRF4, JUN, MALT1, MAP2K6, MAP3K7, MAPK14, MAPK8, MAPK9, MAX, MEF2D, NFATC3, NFKB1, PIK3R1, PTPN18, RAF1, RAPGEF1, RASGRP3, REL, SH3BP2, SOS1, SYK, TEC
Mitochondrial LC-Fatty Acid Beta-Oxidation:WP368	9	52.94117647	2.329848841	ACADM, ACSL1, ACSL3, EHHADH, HADHA, PECR, RPL4, SCP2, SLC25A20
Glycogen Metabolism:WP500	15	45.45454545	2.288141039	AGL, PHKA1, PHKB, PHKG1, PPP2CA, PPP2R2A, PPP2R2C, PPP2R3A, PPP2R5A, PPP2R5C, PPP2R5D, PPP2R5E, PYGB, PYGL, UGP2
Integrated Breast Cancer Pathway:WP1984	25	39.68253968	2.142354953	AHR, BMPR1A, BRCA2, CDC25A, CDC42, CDK7, CERK, DAG1, HDAC1, HMGCR, IRS1, JUN, KRAS, MTOR, MYCBP2, RALGAPA1, RAP1A, RASGRP3, SMEK1, SMEK2, STK11, TSC TSC2, USP38, VEGFA

Supplementary Table S1. The affected genes from exon array analysis of BCAS2-depleted MCF7 cells. RNAs were isolated from MCF7 cells transfected with shBCAS2#1 or pSUPER vector (control) for 48hr. RNA quality was proven by capillary electrophoresis (data not shown) before analyzed by exon arrays. % change is defined by the percentage of alternatively regulated genes in each category when depleting BCAS2 compared with control. The Z-score is defined by calculating the difference between observed and expected number of genes and dividing the difference by the standard deviation of observed number of genes. The data were analyzed by AltAnalyze software from two independent experiments (http://www.altanalyze.org/help.html).

Antibody	Company & Cat. No.	Antibody	Company & Cat. No.
Cre recombinase	Abcam; ab190177	V5	Sigma Aldrich; V8137
NeuN	Millipore; MABN140	β-actin	Sigma-Aldrich; A5441
MAP2	Millipore; MAB3418	BCAS2	Proteintech; 10414-1-AP
Dcx	Millipore; AB2253	β-catenin	BD; 610154
GFAP	Sigma Aldrich; G3893	Dll1	Santa Cruz; sc-9102
IBA-1	GeneTex; GTX101495	anti-mouse IgG	Sigma Aldrich; A9044
Sox2	Abcam; ab79351	anti-Rabbit IgG	Sigma Aldrich; A0545
Phalloidin	Sigma Aldrich; P1951	Flag	BIO-RAD; MCA4764GA
Brdu	Abcam; ab6326	Flag (WB)	Sigma Aldrich; F3040
V5 (WB)	Invitrogen; 46-0705		

Supplementary Table S2. Antibody lists.

PCR Primer	Sequence (5' to 3')	PCR Primer	Sequence (5' to 3')
CU	CAAGAACAGGTATCACTCACGAG	Dll1_R	CCCCAATGATGCTAACAGAAACG
FD	ACATTATAGCGAATCTTTTCCC	GAPDH_F	TGCCAAGGCTGTGGGCAAGG
Cre_F	TCTGATGAAGTCAGGAAGAACC	GAPDH_R	TGTTGGGGGCCGAGTTGGGA
Cre_R	GAGATGTCCTTCACTCTGATT	mBCAS2_F	CCGGATTATTCTGCCTTTGA
β-catenin_F	GCGTGGACAATGGCTACTCAAG	mBCAS2_R	ATGTTCTTTCGCTGCCAGTT
β-catenin_R	GTCATTGCATACTGCCCGTCAA	BCAS2_F	AAAGACTGGCTGCTCGAC
Dll1_F	GGTACTGCGAGGACAATGTGG	BCAS2_R	AGATCCAGCTGTGAGTTG
**qPCR Primer**	**Sequence (5' to 3')**	**qPCR Primer**	**Sequence (5' to 3')**
qβ-actin_F	GTTGTCGACGACGAGCG	qmβ-cateninE3E4_R	TAGCGTCTCAGGGAACATGGC
qβ-actin_R	GCACAGAGCCTCGCCTT	qmβ-cateninI2E3_F	CAATGGGTCATTGTCGTAG
qBCAS2_F	TCCAGCTGTGAGTTGCATGT	qmβ-cateninI2E3_R	TGGCTGACAGCAGCTTTTCTG
qBCAS2_R	CACAGCATGGATGTAATGCC	qDLL1E4E4_F	AACCCAGAAAGACTCATCAGC
qHistone3_F	ATCCGCCGCTACCAGAAGT	qDLL1E4E4_R	AGGGTTGCACACTTTCTCC
qHistone3_R	CCGCTGGAAGGGCAACT	qDLL1E6E7_F	TATCCGCTATCCAGGCTGTC
qβ-cateninE3E4_F	GGATTTTCTCAGTCCTTCACTCAAG	qDLL1E6E7_R	GGGTCACACTCGTCAATCC
qβ-cateninE3E4_R	TGAGCTCGAGTCATTGCATACTGT	qDLL1I3E4_F	GAACCAACCAGGAGCATCTT
qβ-cateninI3E4_F	GCTGAACTGTGGATAGTGAGTGTTG	qDLL1I3E4_R	GTAGGAGTACTTGAGGTCCGT
qβ-cateninI3E4_R	TGAGCTCGAGTCATTGCATACTG	qDLL1I8E8_F	AGCTCTTCACCCTGTTCTAATG
qNanog_F	ATGCGGACTGTGTTCTCTCA	qDLL1I8E8_R	ACTCGAGGTCACTCACAAATG
qNanog_R	GCAATGGATGCTGGGATACT	qGAPDH_F	CATGGCCTTCCGTGTTCCT
qmβ-cateninE3E4_F	GTTGACACCTCCCAAGTCCT	qGAPDH_R	GCGGCACGTCAGATCCA
**RNAi**	**Sequence (5' to 3')**	**shRNA**	**Sequence (5' to 3')**
Ctrl siRNA	TTCTCCGAACGTGTCACGTdTdT	shmB2#1	CTCGACAACCGATTGAA
siβ-catenin	ACCATGCAGAATACAAATGATdTdT	shmB2#2	CCATGGACTATGTAAACTGTT

Supplementary Table S3. Primer and RNAi target sequences.

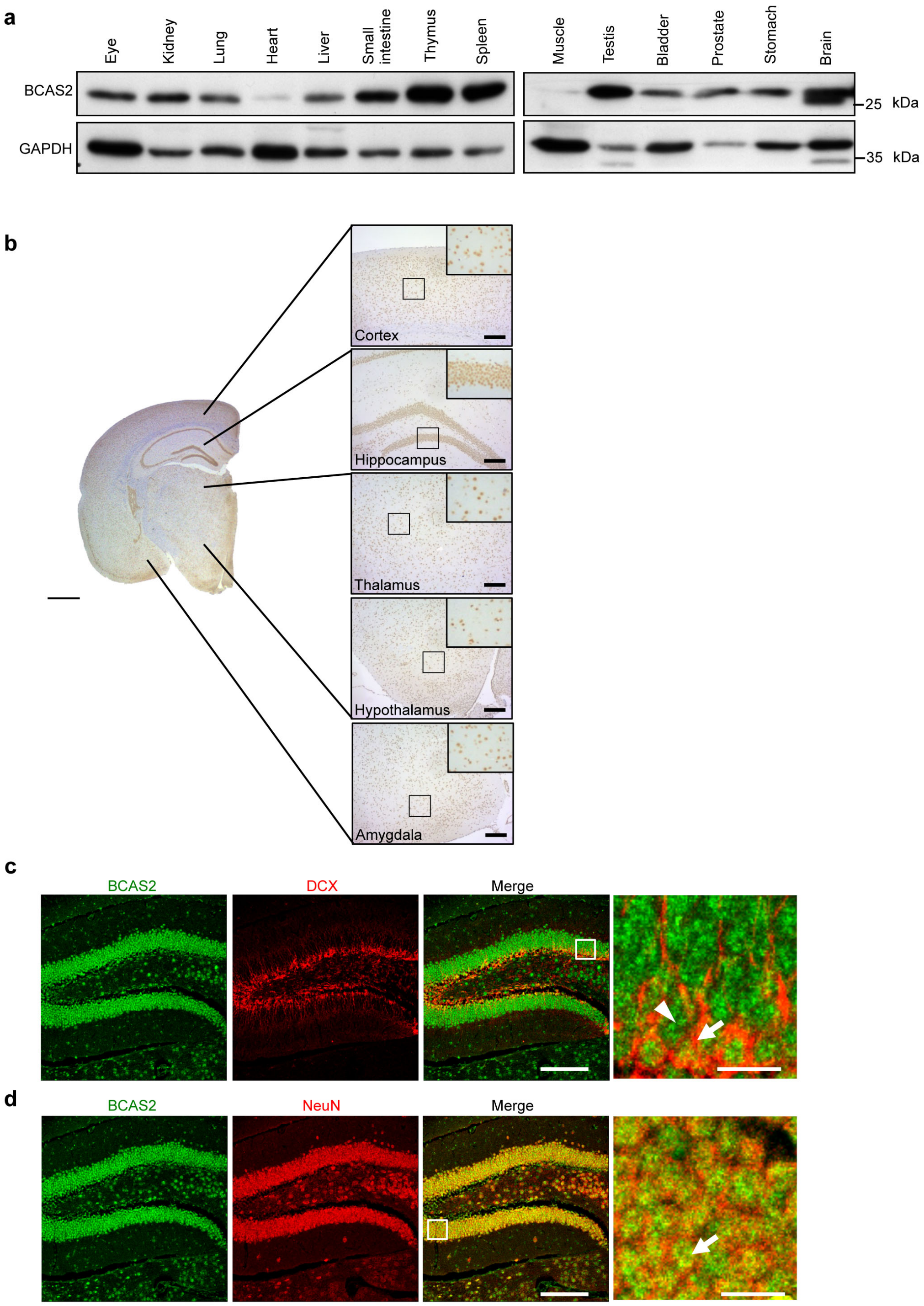

Supplementary Figure S1. Expression of BCAS2 in WT mice. (**a**) Western blot analysis of the BCAS2 protein expression in various tissues at age 12 weeks. (**b**) IHC of the BCAS2 expression in 4-μm forebrain paraffin sections of WT mice at 12 weeks of age with counterstaining by hematoxylin. Scale bar: left: 1 mm, right: 200 μm. (**c**)

BCAS2 is co-expressed with DCX. BCAS2 was expressed in the nucleus (white arrowhead), and DCX was expressed in the cytoplasm of neuroblasts/immature neurons in the inner cell layer of the DG (white arrow). (**d**) BCAS2 co-expression with NeuN in the nuclei of mature neurons in the hippocampus shown by confocal microscopy (white arrow). Scale bars: 200 μm in the main panels and 20 μm in the enlarged panels. Uncropped blots are presented in Supplementary Figure S6.

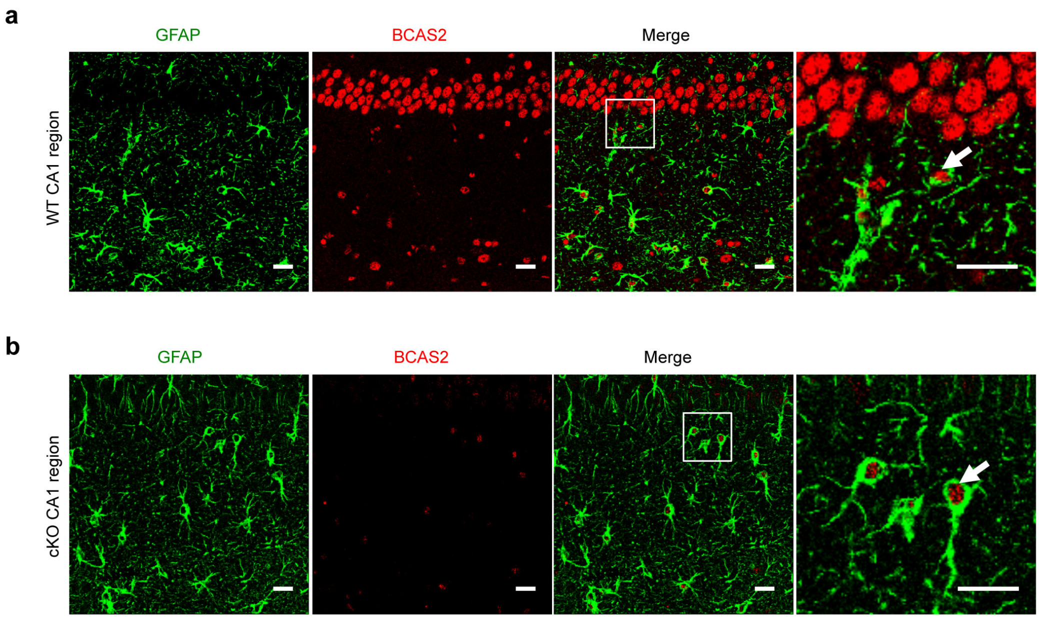

Supplementary Figure S2. BCAS2 can be detected in GFAP astrocytes in both WT and cKO mice. Immunofluorescence analysis of 4-μm forebrain paraffin sections was performed with anti-BCAS2 (red) and GFAP (green) antibodies in coronal sections for the genotypes. The GFAP^{+} astrocytes in CA1 of 12-week-old WT (**a**) and cKO mice (**b**) are indicated by arrows. Images were obtained using confocal microscopy. Scale bars: 20 μm.

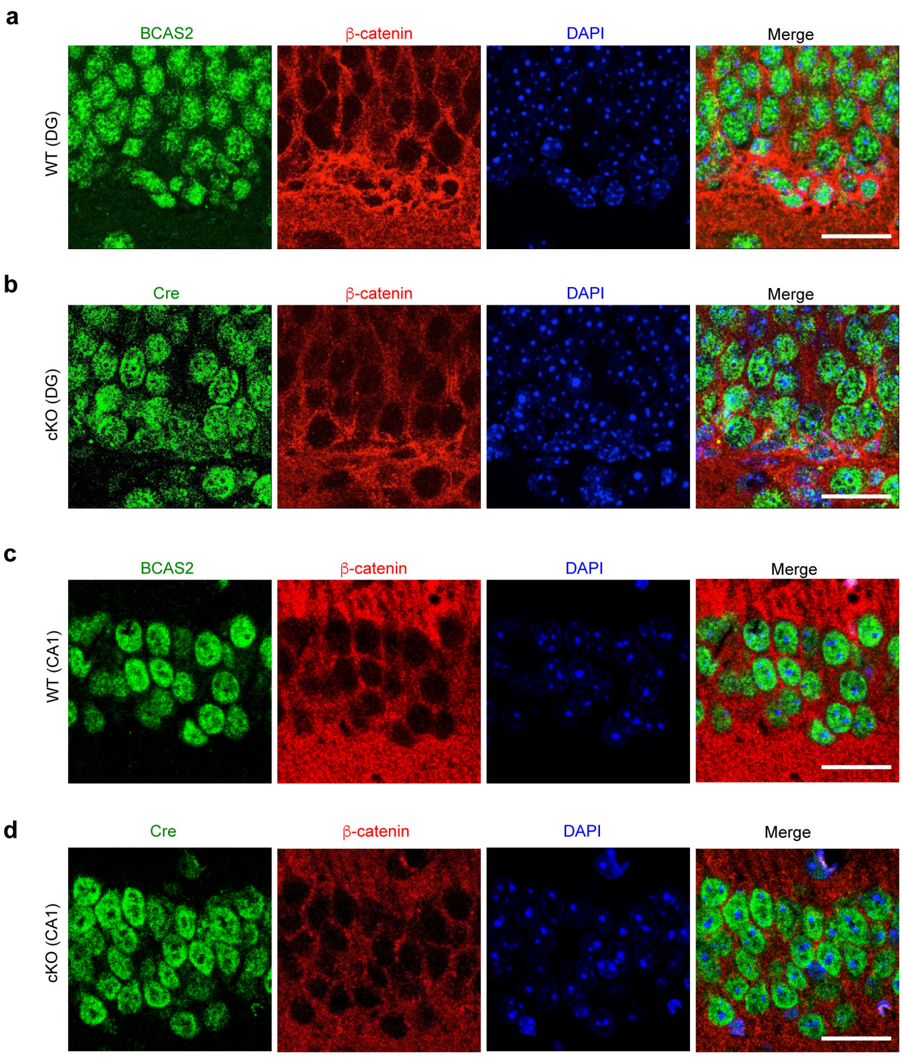

Supplementary Figure S3. BCAS2 knockout resulted in a reduction in β-catenin in the hippocampus. (**a**) Coexpression of BCAS2 and β-catenin in the DG of WT mice. The 4-μm forebrain paraffin sections were examined using IFA with anti-β-catenin and anti-BCAS2 antibodies. Green: BCAS2. Red: β-catenin. (**b**) Lower expression of β-catenin detected in the DG of BCAS2 cKO mice compared to (**a**). (c) Coexpression of BCAS2 and β-catenin in the CA1 of WT mice. (d) Lower expression of β-catenin detected in the CA1 of BCAS2 cKO mice compared to (**c**). Green:

BCAS2 or Cre. Red: β-catenin. Images were obtained using confocal microscopy. Scale bars: 20 μm.

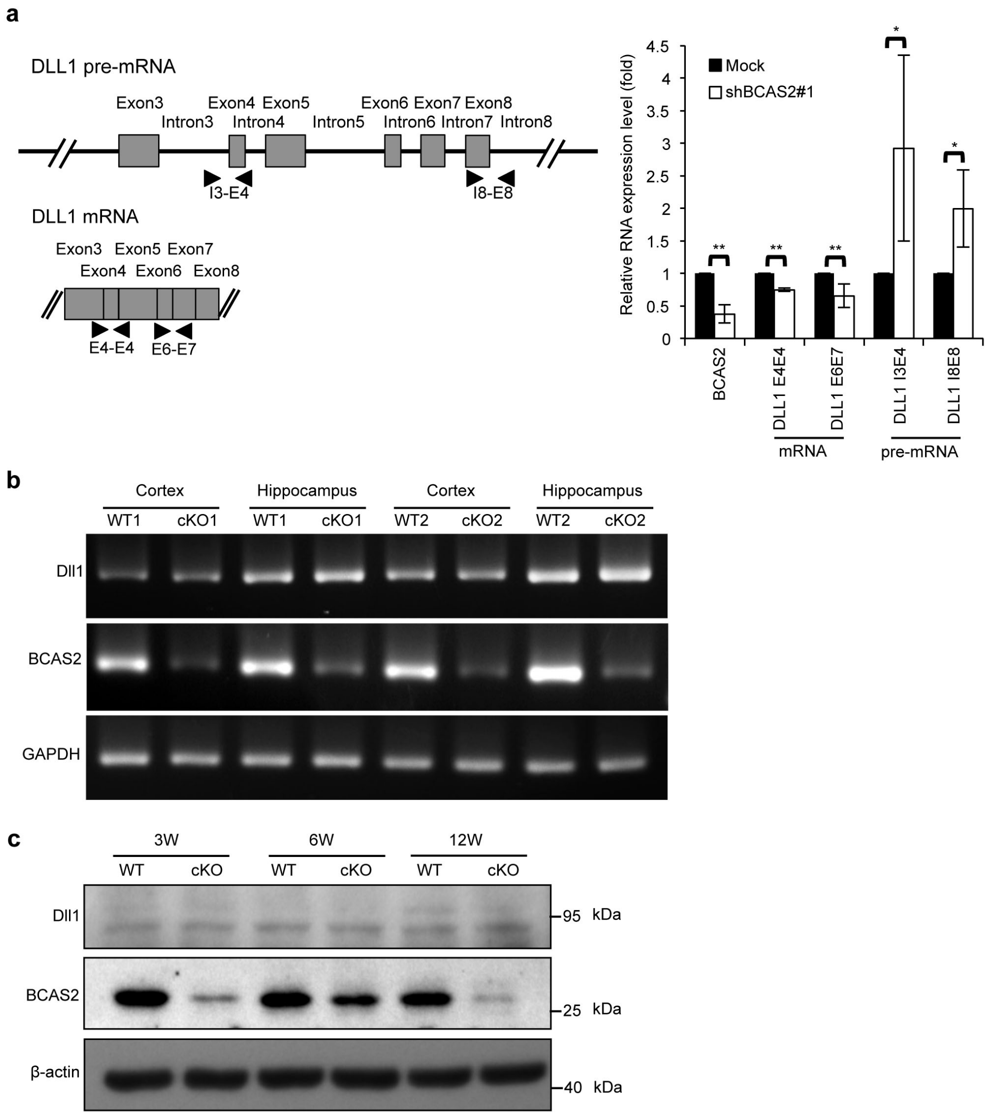

Supplementary Figure S4. BCAS2 regulates the expression of *Delta* in human cell lines but not in mice. (**a**) BCAS2 knockdown causes increased *Delta* pre-mRNA and reduced *Delta* mRNA levels in MCF7 cells. Left: schematic representation of the design of primers for detecting the intron-containing precursor mRNA (upper) and mRNA of *Delta* (lower). Primers, exons and introns are shown by arrowheads, boxes and lines, respectively. Right: quantitative PCR showing the expression of pre-mRNA and mRNA of *Delta*. The data are the mean ± SD relative to controls from 3 independent experiments. *$P < 0.05$, **$P < 0.01$ by Student's *t*-test. (**b**) RT-PCR

analysis of the *Delta* mRNA expression in the hippocampal and cortical tissues of WT and cKO mice (12-week-old). (**c**) Western blot showing the Delta protein expression from the forebrain tissues for each genotype at 3, 6 and 12 weeks of age. Uncropped blots and DNA gels are presented in Supplementary Figure S6.

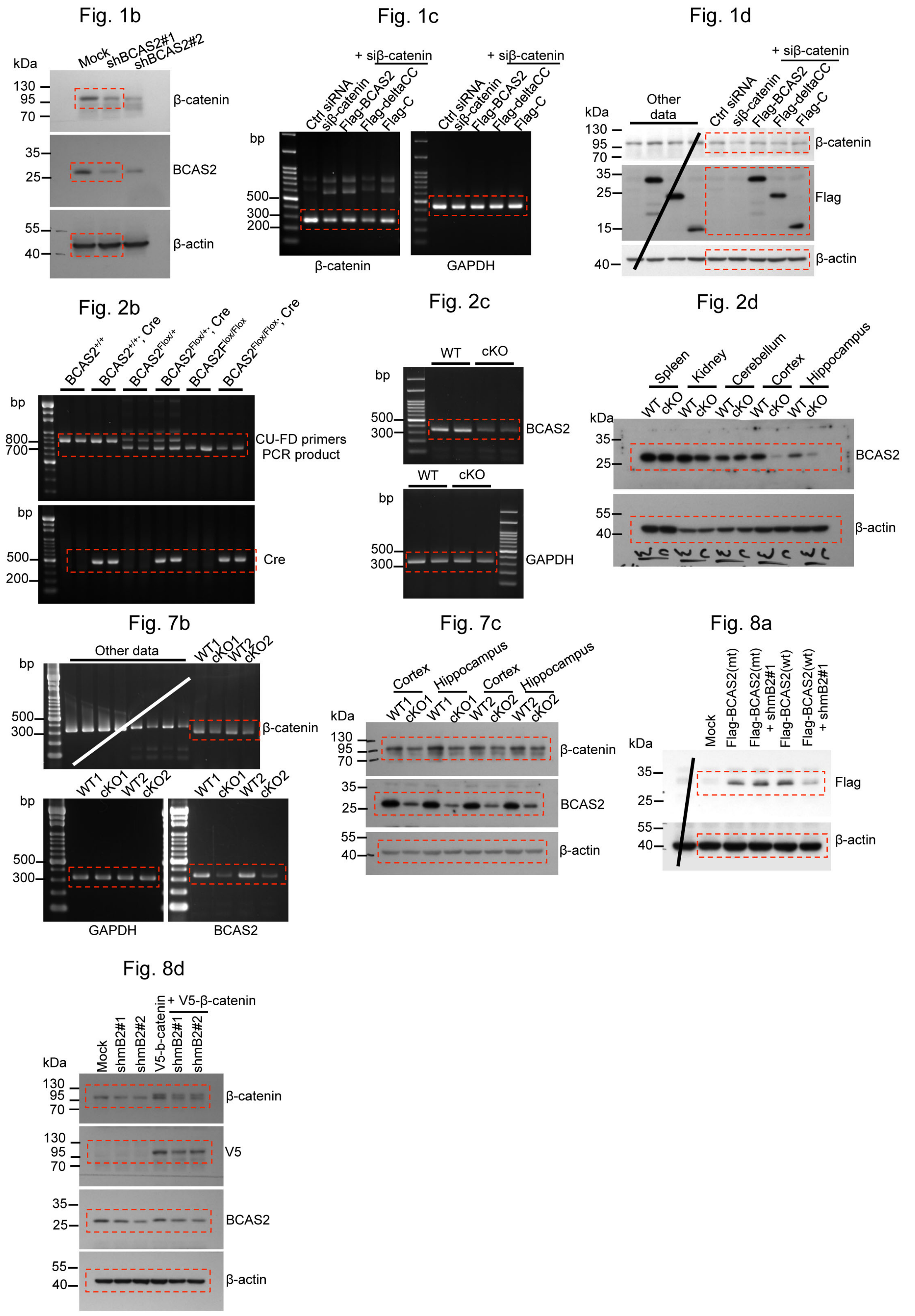

Supplementary Figure S5. Uncropped western blot membranes and DNA gels.

Uncropped original pictures of the western blot and DNA gels presented in the

indicated main figures. The red dotted boxes indicate cropped regions. Western blot membranes were often cut in different pieces according to the molecular weight of the target proteins that were blotted with different antibodies within one membrane separately.

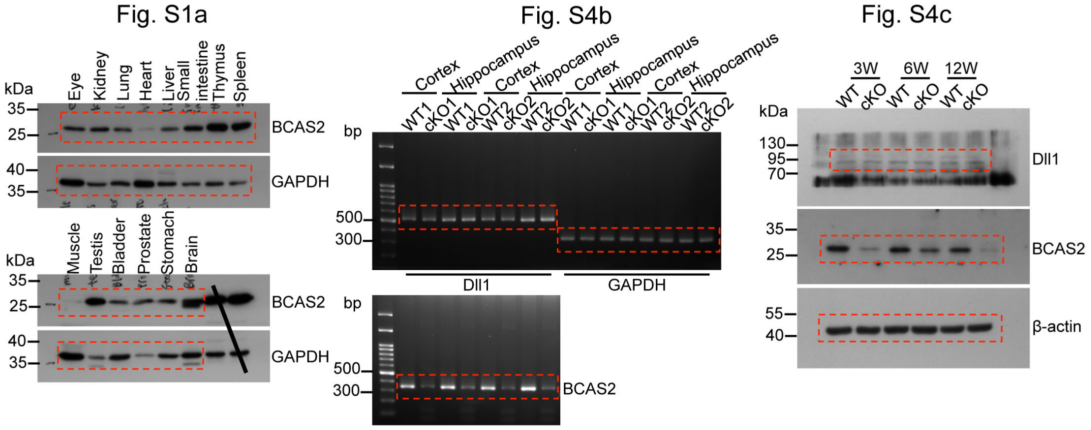

Supplementary Figure S6. Uncropped western blot membranes and DNA gels. Uncropped original pictures of western blot and DNA gels presented in the indicated supplementary figures. The red dotted boxes indicate cropped regions. Western blot membranes were often cut in different pieces according to the molecular weight of the target proteins that were blotted with different antibodies within one membrane separately.
